# A novel recombinant antibody specific to full-length stromal derived factor-1 for potential application in biomarker studies

**DOI:** 10.1371/journal.pone.0174447

**Published:** 2017-04-05

**Authors:** Daniel I. Bromage, Stasa Taferner, Mahesh Pillai, Derek M. Yellon, Sean M. Davidson

**Affiliations:** The Hatter Cardiovascular Institute, University College London, London, United Kingdom; National Cancer Institute, UNITED STATES

## Abstract

**Background:**

Stromal derived factor-1α (SDF-1α/CXCL12) is a chemokine that is up-regulated in diseases characterised by tissue hypoxia, including myocardial infarction, ischaemic cardiomyopathy and remote ischaemic conditioning (RIC), a technique of cyclical, non-injurious ischaemia applied remote from the heart that protects the heat from lethal ischaemia-reperfusion injury. Accordingly, there is considerable interest in SDF-1α as a potential biomarker of such conditions. However, SDF-1α is rapidly degraded and inactivated by dipeptidyl peptidase 4 and other peptidases, and the kinetics of intact SDF-1α remain unknown.

**Methods & results:**

To facilitate investigation of full-length SDF-1α we established an ELISA using a novel recombinant human antibody we developed called HCI.SDF1. HCI.SDF1 is specific to the N-terminal sequence of all isoforms of SDF-1 and has a comparable K_D_ to commercially available antibodies. Together with a detection antibody specific to the α-isoform, HCI.SDF1 was used to specifically quantify full-length SDF-1α in blood for the first time. Using RIC applied to the hind limb of Sprague-Dawley rats or the arms of healthy human volunteers, we demonstrate an increase in SDF-1α using a commercially available antibody, as previously reported, but an unexpected decrease in full-length SDF-1α after RIC in both species.

**Conclusions:**

We report for the first time the development of a novel recombinant antibody specific to full-length SDF-1. Applied to RIC, we demonstrate a significant decrease in SDF-1α that is at odds with the literature and suggests a need to investigate the kinetics of full-length SDF-1α in conditions characterised by tissue hypoxia.

## Introduction

Stromal derived factor-1α (SDF-1α/CXCL12) is a CXC chemokine that is expressed in several tissues in response to hypoxia, via up-regulation of hypoxia inducible fator-1α (HIF-1α).[1–5] For example, SDF-1α is up-regulated in experimental and clinical studies of acute myocardial infarction (MI), wherein it is thought to mitigate adverse ventricular remodelling. Its mechanism of action is reportedly as a chemo-attractant for a variety of cell types expressing its cognate G protein-coupled receptor, CXCR4, including mesenchymal stem cells (MSCs), adipose-derived regenerative cells, c-kit^+^ endogenous cardiac stem cells and T lymphocytes,[6–9] which subsequently have beneficial paracrine effects.[10] It has also been implicated in acute cardioprotection via its binding to myocardial CXCR4 and subsequent activation of the reperfusion injury salvage kinase pathway.[11, 12]

Consequent upon this mechanism, SDF-1α has been evaluated as a potential biomarker in several cardiovascular contexts, including both as a predictor of incident cardiovascular events and of adverse outcome. Specifically, studies have investigated SDF-1α in MI with and without ST segment elevation,[13–16] cardiac surgery,[17] heart failure,[18] patients at increased cardiovascular risk,[19] and in relation to human heart transplantation.[20] Similarly, remote ischaemic conditioning (RIC), a technique of cyclical, non-injurious ischaemia applied to an organ or tissue remote from the heart, has been reported by us and others to increase SDF-1α as measured using commercially available ELISA kits (typically from R&D Systems, UK).[12]

**Fig 1:**
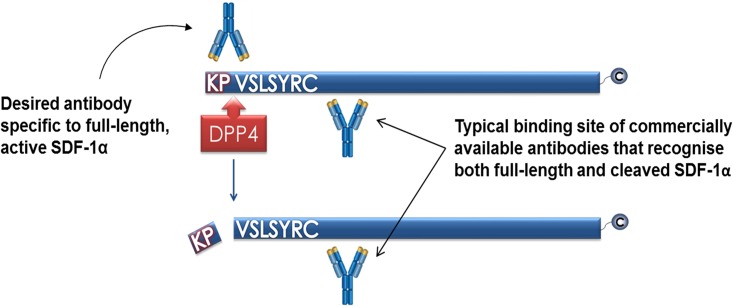
Representation of binding sites of HCI.SDF1α and commercial antibodies on full-length SDF-1α and SDF-1α after cleavage of its N-terminal di-peptide by DPP4.

SDF-1 is cleaved by several peptidases, including leukocyte elastase, matrix metalloproteases 1, 2, 3, 9, 13 and 14, and cathepsin G.[21] However, dipeptidyl peptidase 4 (DPP4), which cleaves SDF-1 at its position 2 proline residue, has been shown to be predominantly responsible and is the focus here.[22] Cleavage reduces the affinity of SDF-1 for CXCR4, rendering it inactive.[23] Currently, commercial antibodies have different affinities towards intact SDF-1α (consisting of amino acids 1–67) and DPP4-truncated SDF-1α (amino acids 3–67),[21, 24] and the kinetics of full-length, active SDF-1α in response to tissue hypoxia are unknown (Fig 1). Analysis is complicated by the absence of a method to specifically quantify active SDF-1α in plasma. We therefore aimed to develop a method to investigate intact SDF-1α, using RIC as an example.

## Materials & methods

### Ethics statement

All procedures were performed with Local Research Ethics Committee approval at the Hatter Cardiovascular Institute, University College London (12/0448). Experiments were conducted according to the principles expressed in the Declaration of Helsinki. Volunteers gave informed consent. All use of animals was in accordance with the United Kingdom (Scientific Procedures) Act 1986. The experiments were prospectively approved by the UK Home Office animal research ethics committee under Project Licence number PPL 70/8556, (“Protection of the Ischaemic and Reperfused Myocardium”) issued to Prof. Derek Yellon in 2015. Adult male Sprague-Dawley rats weighing 200–250 g (Harlan Laboratories, UK) were euthanized by terminal anaesthesia with 100 mg/kg pentobarbitone sodium administered IP.

### Antibody characterisation

A custom human IgG1 bivalent mini-antibody, with embedded FLAG octapeptide and polyhistidine (His6) tags, was identified from the HuCAL^®^ (Human Combinatorial Antibody Library) phage-display library containing several billion, distinct, fully human antibodies.[25, 26] The library was screened using positive selection for binding to the peptide KPVSLSYR-Ttds-C derived from the N-terminus of full-length SDF-1(1–8) and negative selection for binding to the peptide VSLSYR-Ttds-C derived from the N-terminus of cleaved SDF-1(3–8) (AbD Serotec, UK). These sequences are common to all isoforms of SDF-1 (alpha-zeta), although SDF-1α is predominant.[27] From the 12 initially identified clones, HCI.SDF1 was identified as having the most specific and robust binding to full-length SDF-1 without any binding to cleaved SDF-1 or to glucagon-like peptide-1 (GLP-1), an unrelated protein that is also cleaved by DPP4.

### HCI.SDF1 kinetics

To ascertain the association and dissociation kinetics of the antibody, a label-free sensor system (Octet Red, ForteBio, UK) was used with SDF-1α, according to the manufacturer’s instructions. HCI.SDF1 was diluted to 5 μg/ml in 0.1% BSA-PBS supplemented with 0.02% Tween^®^20 (Sigma-Aldrich, UK). The antibody was immobilised on Amine Reactive Second-Generation (AR2G) Biosensors (ForteBio, UK) at 30°C for 180 s before being dipped into the same buffer containing a range of concentrations of SDF-1α (1 μg/ml with serial twofold dilutions) for 600 s. The sensors were then returned to the buffer for the dissociation reaction, for 600 s. For each concentration of SDF-1α, the kinetic curves for association and dissociation were obtained and used to calculate a global K_D_ with the analysis programme provided by the manufacturer (Octet DataAnalysis v9.0.0.12).

### HCI.SDF1 ELISA

**Fig 2:**
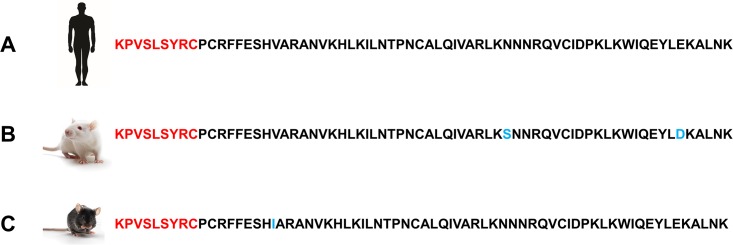
SDF-1α sequence according to species. Alignment of SDF-1α from humans, rats and mice demonstrates the N-terminal sequence is highly conserved between species. Accession numbers NP_001171605.1, NP_001029055.1 and NP_001012495.1 were used, respectively. Sequences in red represent the sequence of interest used to generate HCI.SDF1. Blue residues represent differences in the primary sequence between species; note that none of these are at the N-terminal end.

HCI.SDF1 was optimised as the capture antibody in a sandwich ELISA for measuring full-length, un-cleaved SDF-1α, with detection by a biotinylated polyclonal goat IgG against the alpha isoform of human and mouse SDF-1 (BAF310, R&D Systems, UK) and streptavidin-HRP (R&D Systems, UK). A standard curve was generated using recombinant human (rh) SDF-1α (R&D Systems, UK) diluted in BUF037A, a diluent with mammalian serum proteins to mimic the target analyte recovery profile of human plasma (AbD Serotec, UK). Rat SDF-1α is 97% identical to the human protein overall, and at the N-terminus is 100% identical to both human and mouse SDF-1α (Fig 2). The optical density of each well was measured using a 96 well microplate reader (FLUOstar Omega, BMG Labtech, Germany) set to 450 nm. All analyses, including samples and standards, were performed in triplicate to minimise intra-assay variability, and baseline corrected.

In addition, to permit comparison of our novel assay with commercially available antibodies, MAB350, a monoclonal mouse IgG against mouse and human SDF-1α, was substituted as the detection antibody using the same assay protocol. This antibody was selected based on the capture antibody used in a commercially available and widely used ELISA for SDF-1α (Quantikine^®^ ELISA for Human CXCL12, R&D Systems, UK).

To ascertain the sensitivity of each respective assay to intact and cleaved forms of SDF-1α, human platelet-free plasma was spiked with 3,000 pg/ml intact rhSDF-1α and incubated with 800 μM rhDPP4 (R&D Systems, UK) in Tris buffer for 2 h at 37°C prior to termination of DPP4-mediated cleavage with 100 mM Sitagliptin (Merck, US) and mixing known proportions with intact SDF-1α.

### Western blotting

Western blot analysis was performed on samples of rhSDF-1α (1–50 ng) denatured in Laemmli gel sample buffer. HCI.SDF1 was used at a dilution of 1:400 and detected using 1:5000 goat anti-human IgG Fab-FITC (AbD Serotec, Oxon, UK).

### Animal experiments

Adult male Sprague-Dawley rats weighing 200–250 g (Harlan Laboratories, UK) were anesthetised with 100 mg/kg intra-peritoneal pentobarbitone sodium. Animals underwent orotracheal intubation and ventilation using allometrically adjusted tidal volume and ventilation rates, and supplementary oxygen. Core body temperature was monitored via a rectal temperature sensor and maintained at 36.5±0.5°C by adjustment of a homeothermic heat mat (Kent Scientific, USA). ECG was recorded throughout using PowerLab 4/25 and Animal Bio Amp coupled to Chart 7 (AD Instruments, UK).

### Remote ischaemic conditioning protocol

**Fig 3:**
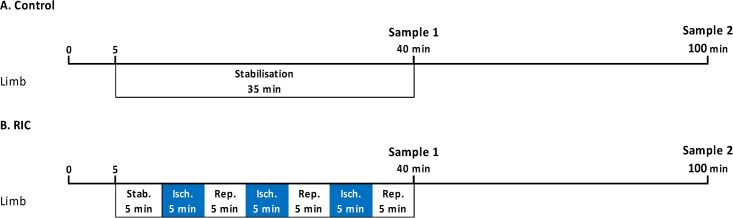
*In vivo* rat protocols. Remote ischaemic conditioning (RIC) was applied to the rat hind limb in the following groups: A) Control: 35 min stabilisation; B) RIC: 5 min stabilisation, 3 cycles of 5 min ischemia and 5 min reperfusion. Blood samples were taken either immediately after RIC (sample 1) or 60 min thereafter (sample 2).

**Fig 4:**
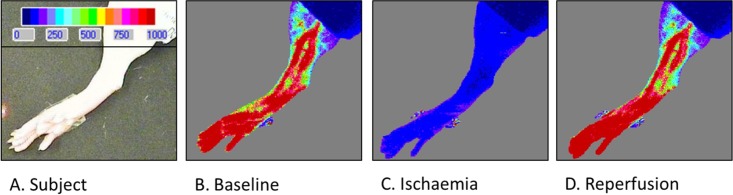
Laser Doppler blood flow assessment in the subject hind limb during RIC and reperfusion. Panel A shows a colour photograph of the subject hind limb. Panel B demonstrates complete cessation of blood flow during RIC ischaemia (C) and restoration after reperfusion (D).

Animals were randomly assigned to either control or RIC groups. RIC was applied using an inflatable cuff (Hokanson, USA) around the right hind limb, which was inflated to 200 mmHg using a manual sphygmomanometer. 3 cycles of 5 min ischaemia and 5 min reperfusion was applied (Fig 3). Ischaemia was indicated by cyanosis of the right hind limb and confirmed by global flow assessment with a high resolution laser Doppler imager (Moor Instruments, UK). Representative images are shown in Fig 4. Control animals underwent a sham procedure, including identical anaesthetic time. Samples were collected either immediately after RIC or 1 h later (Fig 3), as described below.

### Blood collection for SDF-1α and DPP4 measurement

4.5 ml of blood was collected on ice into tubes containing 250 μl of 1 mM Sitagliptin to inhibit DPP4 and prevent degradation of intact SDF-1α during sample preparation,[21] and 0.5 ml 10x citrate buffer to prevent coagulation. Samples for DPP4 measurement were collected without Sitagliptin. Aspiration of blood was performed by right ventricular puncture using a wide-bore 19G x 1.5” needle to avoid platelet activation and subsequent release of SDF-1α. Samples were immediately centrifuged at 1,600 g for 20 min then 10,000 g for 30 min to obtain platelet-free plasma and frozen at -80°C. After all experimental samples were collected they were defrosted at room temperature and assayed immediately.

To investigate the effect of sample preparation on the measurement of SDF-1α with our novel assay, separate blood samples were collected in the same way but only centrifuged at 1,600 g for 20 min to obtain unfractionated plasma. Further samples were allowed to clot at room temperature for 20 min before centrifuging at 1600 g for 10 min to obtain serum.

### Human experiments

Six healthy male volunteers participated in the study after giving written informed consent. None were taking any regular medication. Each volunteer was subjected to RIC, consisting of 3 cycles of 5 min right upper limb ischaemia by inflation of a manual sphygmomanometer to a pressure of 200 mmHg. Ischaemia was confirmed by absence of a pulse, and presence of pallor and paraesthesia. Samples were collected into tubes containing Sitagliptin either immediately after RIC or 1 hour later via ipsilateral antecubital fossa venepuncture (Fig 3). Experiments were all conducted at the same time of day.

### Statistical methods

On the basis of our previous findings using an *in vivo* model of RIC, we calculated that a 20% increase in plasma SDF-1α at a significance level of 0.05 and a power of 0.8 would require 4 animals in each group.[28] Results of animal experiments were compared using the Student t test for 2 groups of continuous variables and analysis of variance (ANOVA) and Tukey’s Multiple Comparison Test for 3 or more groups. Data is presented as mean ± SEM. Statistical significance was reported if P<0.05 using the following nomenclature: *P<0.05, **P<0.01 and ***P<0.001. Results where P>0.05 were reported as non-significant (NS). Sample values were baseline corrected and linear (HCI.SDF1) or nonlinear using log(agonist) versus response (MAB350) regression of standard values was performed with GraphPad Prism^®^ version 5.00 for Windows (California, USA).

## Results

### Identification and characterisation of an ELISA for HCI.SDF1a

A custom, human, monoclonal IgG1 antibody was identified from the HuCAL^®^ library, which contains more than 45 billion functional human antibody specificities.[25, 26] The phagemid library was screened for binding to a peptide derived from full-length SDF-1 without binding to a peptide derived from cleaved SDF-1. HCI.SDF1 was the clone that best satisfied these criteria and was optimised as the capture antibody in a sandwich ELISA for full-length SDF-1α. The intra- and inter-assay variations of our ELISA for intact SDF-1α were 2.7% and 7.1% respectively, which are below the recommended maximum threshold of 10%. The limit of detection (LoD) and limit of quantification (LoQ) in platelet-free plasma were 156 pg/ml and 312 pg/ml, respectively.

### The antibody HCI.SDF1 has comparable binding kinetics to MAB350

**Fig 5:**
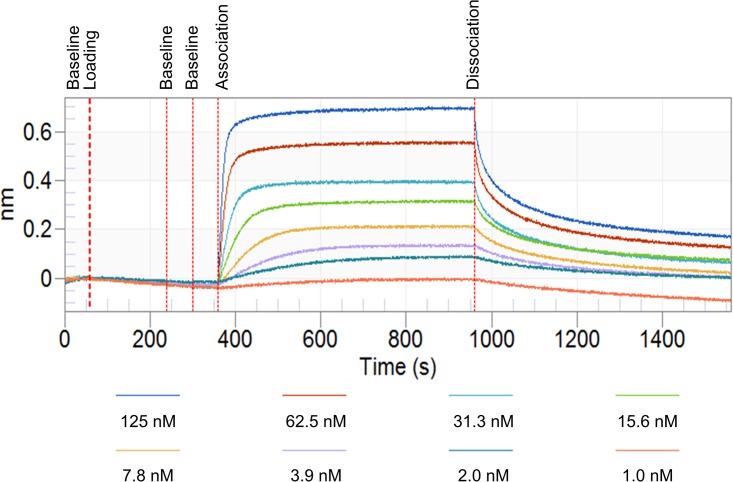
Representative HCI.SDF1 and MAB350 binding kinetics data. Representative data obtained from label-free sensor evaluation of HCI.SDF1 and MAB350 with Amine Reactive Second-Generation (AR2G) Biosensors.

The equilibrium dissociation constant (K_D_) between the recombinant HCI.SDF1 antibody and SDF-1α was measured using the Octet Red label-free sensor system. Concentrations of 0.98 to 125 nM SDF-1α were measured in parallel wells of a 96 well plate. Curve fits were generated and a K_D_ calculated for both HCI.SDF1 (1.4 x 10^−8^ ± 3.5 x 10^−9^ M) and MAB350 (8.0 x 10^−8^ ± 7.0 x 10^−9^ M), which were not significantly different (n = 3 independent experiments), and were within the typical range of affinities of monoclonal antibodies. A representative image from the binding experiment is shown in Fig 5.

### HCI.SDF1a is more sensitive to intact SDF-1α

**Fig 6:**
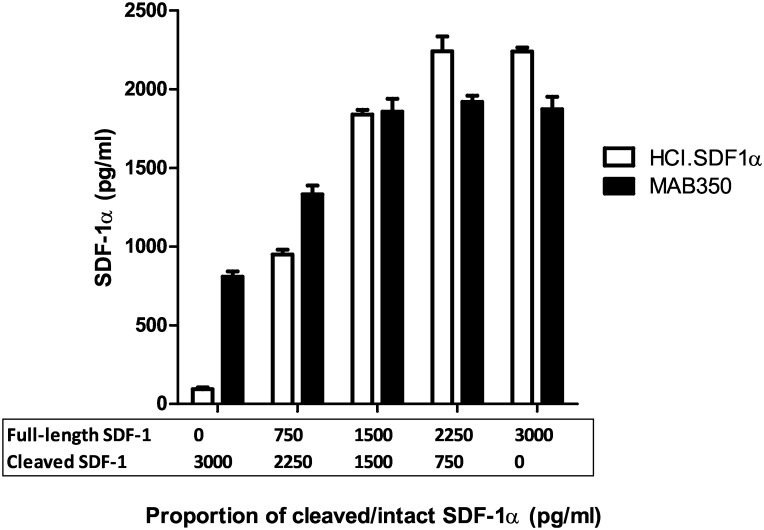
ELISA for intact and cleaved SDF-1α using HCI.SDF1 and MAB350. An ELISA assay demonstrates that HCI.SDF1 is specific for active SDF-1α, with very low signal in the absence of intact SDF-1α, whereas there is substantial residual signal in the absence of intact SDF-1α when using the commercial antibody MAB350.

By assaying different proportions of intact and cleaved SDF-1α we were able to demonstrate that an ELISA using HCI.SDF1 as the capture antibody is specific for full-length SDF-1α, whereas commercial assays report a combination of intact and cleaved SDF-1α (Fig 6), making the results with commercial assays difficult to interpret.

**Fig 7:**
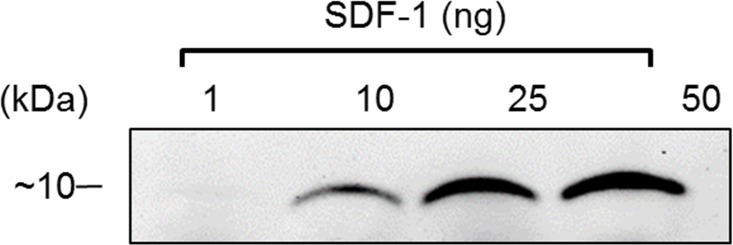
HCI.SDF1 used for Western blotting. Western blots demonstrated a concentration-signal relationship when HCI.SDF1 was used to detect varying concentrations of rhSDF-1α.

Further analysis demonstrated that HCI.SDF1 is able to detect denatured SDF-1α and is therefore suitable for Western blot applications (Fig 7).

### Serum preparation dramatically increases SDF-1α levels

**Fig 8:**
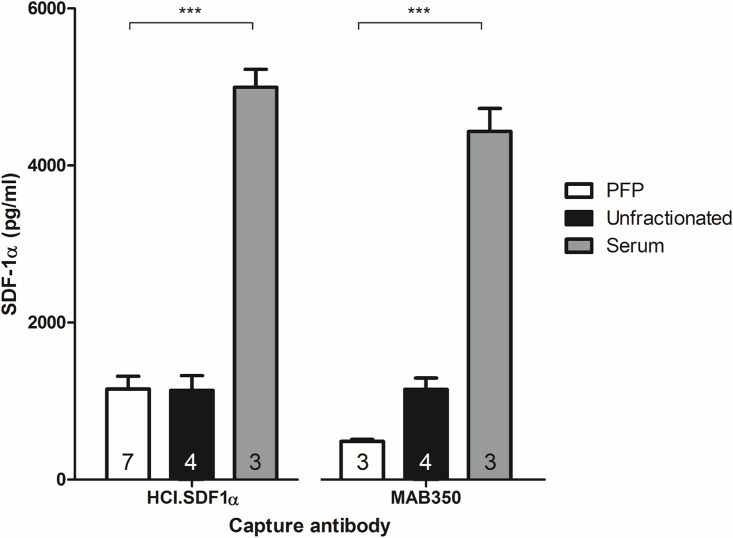
The effect of sample type on SDF-1α concentration. Serum samples, collected by allowing whole blood to clot for 10 min prior to centrifuging to remove the clot, had significantly more active SDF-1α than either platelet-free (PFP) or unfractionated plasma samples that were collected into a citrated tube and prepared by differential centrifugation, when measured using either capture antibody as indicated.

The effect of blood sample preparation on SDF-1α levels was determined using rat platelet-free plasma compared to serum and plasma from which platelets had not been removed (unfractionated plasma). SDF-1α levels were significantly higher in serum samples than either platelet-free or unfractionated plasma samples, when measured by ELISA with either HCI.SDF1 (5000 ± 200 pg/ml, 1150 ± 160 pg/ml & 1140 ± 190 pg/ml, respectively, n = 3–7, P<0.001) or a commercial assay (4400 ± 300 pg/ml, 490 ± 30 pg/ml & 1150 ± 140 pg/ml, respectively, n = 3–4, P<0.001; Fig 8). When serum samples were taken from animals with and without RIC, there was no significant difference between control and RIC groups. Therefore, platelet-free plasma samples were used in all subsequent analyses.

### Plasma levels of full-length SDF-1α decrease after RIC

**Fig 9:**
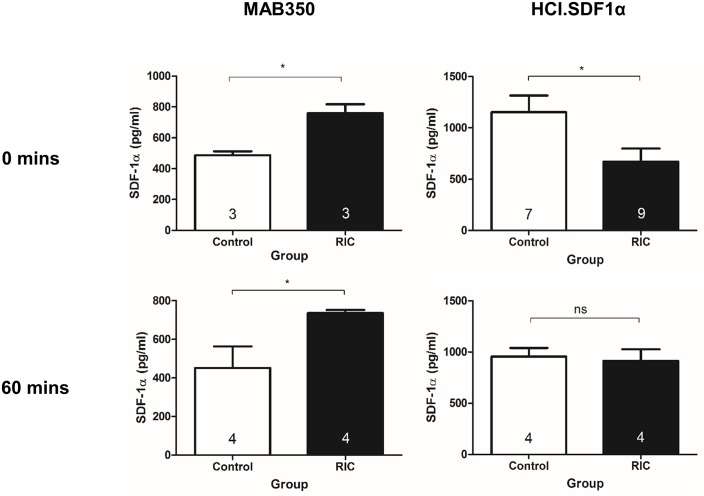
Effect of RIC on intact and cleaved SDF-1α. Following RIC, ELISA using commercial antibodies detects an increase in plasma SDF-1α levels whereas HCI.SDF1α, which is more specific for full-length SDF-1α, measures a decrease immediately after RIC that is restored to baseline by 1 h.

An ELISA using MAB350 (R&D Systems, UK), a monoclonal mouse IgG against mouse and human SDF-1α that is used in most reported studies of SDF-1α replicated our previously reported finding that SDF-1α is significantly increased by an RIC stimulus in rats,[12] and this difference was still present 1 h after the RIC protocol (Fig 9). However, when measured using HCI.SDF1, levels of full-length SDF-1α were unexpectedly found to significantly decrease immediately after RIC (670 ± 130 pg/ml vs. 1150 ± 160 pg/ml, n = 7–9, P<0.05) although they normalised by 1 h (910 ± 110 pg/ml vs. 950 ± 90 pg/ml, n = 4, P = NS).

**Fig 10:**
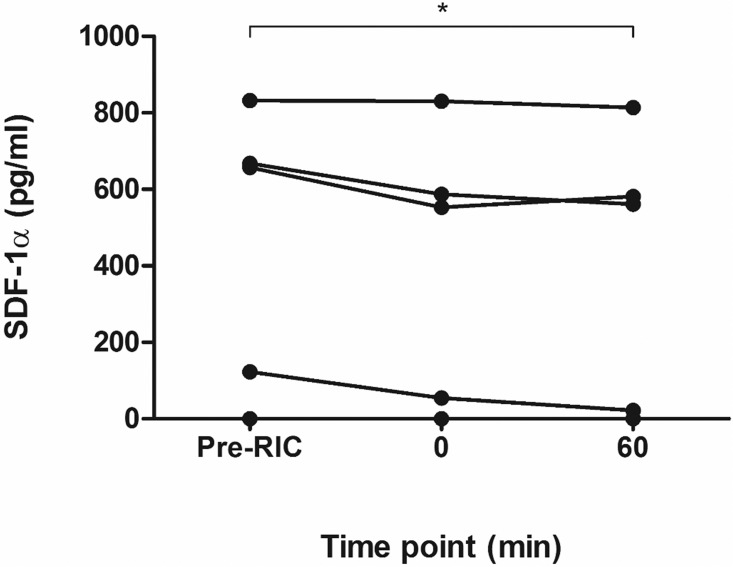
Effect of RIC on intact SDF-1α in humans. Plasma levels of full-length SDF-1α (quantified using HCI.SDF1α ELISA), decreased significantly in healthy male volunteers subjected to RIC.

To confirm our findings in humans, we repeated the RIC protocol on the upper limb of 6 healthy human volunteers and, despite significant inter-individual variability, found a similar trend towards a reduction in active SDF-1α (pre-RIC 380.0±154.9 pg/ml; 0 min 337.5±148.3 pg/ml; 60 min 329.8±148.7 pg/ml, P for trend <0.05, Fig 10).

## Discussion

Several studies have associated increased SDF-1α with both increased risk of incident cardiovascular events and subsequent adverse outcome. For example, in Framingham Heart Study patients, circulating SDF-1α was significantly associated with both new-onset heart failure and all-cause mortality in a multi-variable regression model.[19] SDF-1α was not associated with MI in this study, although genome-wide association studies (GWAS) have identified SDF-1α as a risk locus for both coronary artery disease and MI.[29] Furthermore, in patients with chronic kidney disease, who are at high risk for cardiovascular events, SDF-1α was significantly associated with MI and all-cause mortality.[30] Circulating SDF-1α has also been associated with worse left ventricular (LV) function in patients hospitalised with heart failure.[18]

With respect to the prediction of adverse sequelae after cardiovascular events, elevated SDF-1α has been reported after MI in several studies. [13–16] In ST segment elevation MI (STEMI), higher SDF-1α levels predicted worse LV performance, Killip score and 30 day mortality.[13] SDF-1α levels have also been used to predict adverse events in stable patients with a history of MI [15] and patients with non-STEMI, where SDF-1α levels were an independent predictor of death, MI and heart failure.[16]

Several studies have recorded increased SDF-1α following RIC, as measured with commercially available ELISA kits. For example, Jiang *et al*. demonstrated increased serum and myocardial SDF-1α after a remote ischaemic postconditioning protocol consisting of clamping the infra-renal abdominal aorta for 4 cycles of 5 min ischaemia and 5 min reperfusion. SDF-1ɑ levels peaked at 1 hour, with an 80±5% increase (P<0.001) compared to control (sham surgery) animals.[31] Interestingly, they measured SDF-1α in serum samples, which our findings indicate would result in artefactually high levels of SDF-1α compared to plasma. In a similar experiment, Kamota *et al*. conferred RIC using repetitive occlusion of the abdominal aorta in mice prior to ischaemia-reperfusion of the left anterior descending (LAD) coronary artery territory. Using a standard ELISA, they measured 4-fold levels of SDF-1α after 1 hour in platelet-poor plasma samples.[32] Our group also showed that circulating SDF-1α levels were altered in rats subjected to 3 cycles of 5 min hind-limb ischaemia and reperfusion, increasing by 50% in platelet-free plasma samples to a peak of 890±70 pg/ml.[12]

However, not all studies have reported increased SDF-1α in conditions characterised by tissue hypoxia. Fortunato *et al*. showed a reduction in serum SDF-1α in patients after MI, although higher levels were associated with more adverse events,[14] and Damas *et al*. demonstrated a significant reduction of plasma SDF-1α in patients with stable and unstable angina compared to healthy controls.[33] The investigation of SDF-1α as a biomarker is confounded because the aforementioned studies use the commercially available ELISA from R&D Systems that employs the monoclonal mouse IgG, MAB350. The present study shows that this antibody has different affinity towards full-length and DPP4-cleaved SDF-1α. Baerts *et al*. also observed a significant reduction in immunoreactivity after they measured DPP4-cleaved, recombinant SDF-1α using several different commercial ELISA kits (including R&D Systems, Raybiotech and Peprotech).[11] We observed a partial, but incomplete, loss of signal in an ELISA assay using MAB350 following complete cleavage of SDF-1α with DPP4. This might be explained by altered binding affinity of MAB350 or of the polyclonal detection antibody (BAF310) used in this sandwich ELISA format after cleavage of SDF-1α. As loss of the N-terminal Lys^1^ is known to confer a complete loss of bioreactivity,[23] the incomplete detection of SDF-1α by commercial antibodies confounds interpretation of both basic and clinical studies of SDF-1α that have used commercially available ELISA assays.

Accurate measurement is crucial given the growing interest in SDF-1α both as a potential biomarker and therapy for ischaemic cardiomyopathy.[34–38] Baerts *et al*. recognised that *ex vivo* truncation of SDF-1α significantly affects plasma SDF-1α measurement.[21] Using tubes treated with DPP4 inhibitors they collected plasma samples from patients with heart failure and associated SDF-1α level with heart failure severity, according to ejection fraction. The present study goes further and we report for the first time the application of a novel antibody against the N-terminal Lys^1^, that has similar binding kinetics to commercially available antibodies, in an immunoassay that is specific for intact, active SDF-1. This antibody consequently has greater utility in the investigation of SDF-1α in a variety of settings, when combined with a detection antibody specific to the alpha isoform. Furthermore, as HCI.SDF1 is a recombinant antibody, it is consistent with a reductionist approach to animal experimentation.[39]

In an interesting approach to this problem, Wang *et al*. recently developed a method to quantify intact and cleaved SDF-1α using mass spectrometry.[40] They reported a LoQ of 20 pmol/L to 20 nmol/L in human plasma and, importantly, they demonstrated the differentiation of intact from cleaved SDF-1α to be critical in the investigation of biomarkers of DPP4 efficacy. Using their assay, they report circulating SDF-1α in mice and primates to be in the range detectable by HCI.SDF1 (760 pg/ml and 310 pg/ml, respectively), although they did not report baseline levels in humans. The novel assay described in the present paper has the added advantage of being easy and relatively cost-effective to perform.

Using HCI.SDF1, we first demonstrated that SDF-1α levels are dramatically increased in serum, compared to platelet-free and platelet-poor plasma samples. SDF-1α is known to be contained in platelet granules and be expressed upon platelet activation,[41] as occurs during the preparation of serum samples. The observations we made on changes in SDF-1α levels could not be detected when using serum samples, due to this high background level of SDF-1α. It is therefore important to ensure correct sample preparation when measuring SDF-1α. Published results using existing commercially available assays for SDF-1α are highly variable. Examination of the methods used in these studies reveals highly variable protocols, which include the analysis of both serum and plasma samples.[13, 42, 43]

As an example, we have challenged the existing paradigm that SDF-1α is up-regulated in response to RIC, finding instead a significant decrease in active SDF-1α after RIC. Combined with our previous observation that SDF-1α levels appear to increase when measured with commercial assays, this would suggest that there is, in fact, an increase in cleaved, inactive SDF-1α. To confirm this unanticipated finding we repeated the study in healthy human volunteers and observed a similar decrease in SDF-1α levels after RIC, despite there being significant variability in baseline SDF-1α measurements between individuals. Previously, circulating SDF-1α variability has been associated with various factors including waist circumference,[44] time of day and acute stress.[45] We did not find any significant association between waist circumference and baseline SDF-1α; all experiments were performed at the same time of day to account for SDF-1α circadian rhythm; and no major difference in stress levels of the subjects was apparent, although this was not specifically controlled for.

HCI.SDF1 also has potential value in the investigation of other isoforms of SDF-1. All isoforms are known to agonise CXCR4 but have distinct properties. For example, SDF-1β is more resistant than SDF-1α to proteolysis.[46] Interestingly, expression of SDF-1ɣ has recently been reported in less vascular organs that are susceptible to infarction, including the heart.[46] However, the predominant and best described isoform is SDF-1α.[27]

## Conclusions

SDF-1α is ostensibly increased by RIC, a manoeuvre that has been associated with acute and chronic cardioprotection. However, by using an antibody specific to the intact, active form of SDF-1α we have shown that RIC reduces circulating SDF-1α, which we hypothesise is due to its cleavage, a finding we replicated in both rats and humans. This has implications for the increasing number of studies exploring the potential of SDF-1α as a biomarker in a range of cardiovascular contexts. Further studies are necessary to fully elucidate the value of HCI.SDF1 as a biomarker in relation to clinical phenotypes.

## Supporting information

S1 Fig Fig 6 showing individual data points behind means. (TIF)

S2 Fig Fig 8 showing individual data points behind means. (TIF)

S3 Fig Fig 9 showing individual data points behind means. (TIF)
