# Supplementary figures and images for: A novel recombinant antibody specific to full-length stromal derived factor-1 for potential application in biomarker studies
Source: PLoS One. 2017 Apr 5;12(4):e0174447. doi: 10.1371/journal.pone.0174447 (PMC5381782; doi:10.1371/journal.pone.0174447)

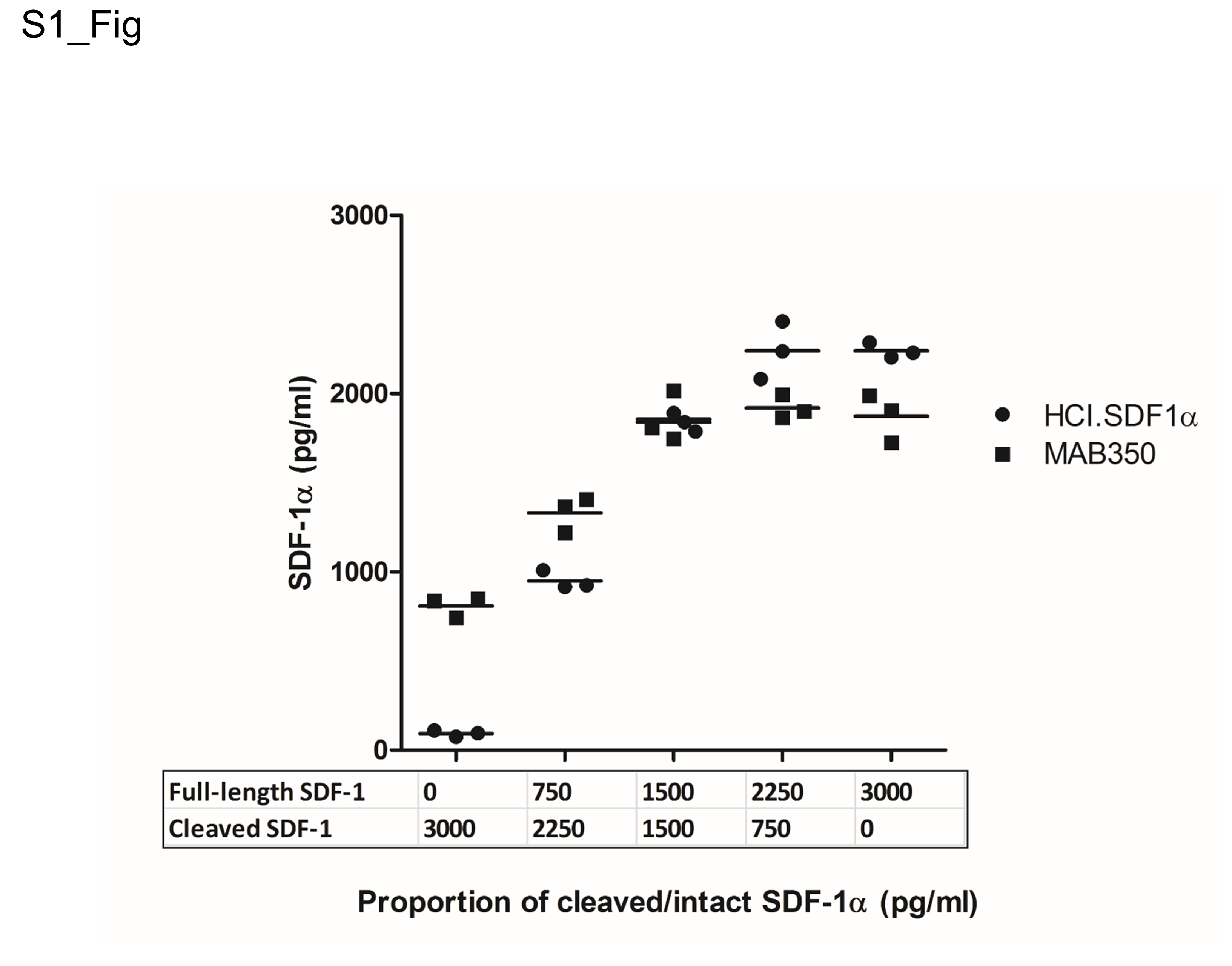

Supplement: S1 Fig — (TIF) [file pone.0174447.s001.tif]

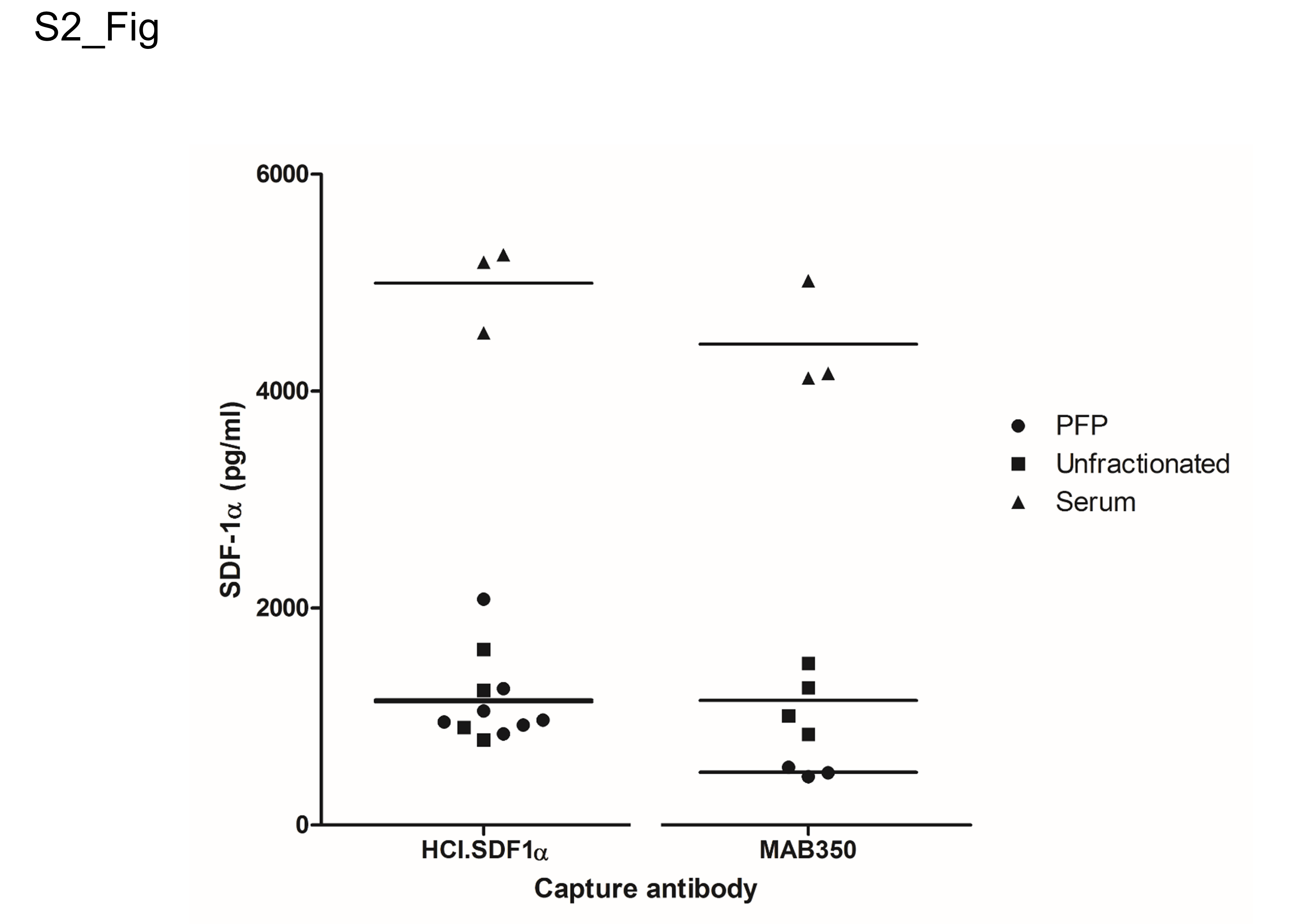

Supplement: S2 Fig — (TIF) [file pone.0174447.s002.tif]

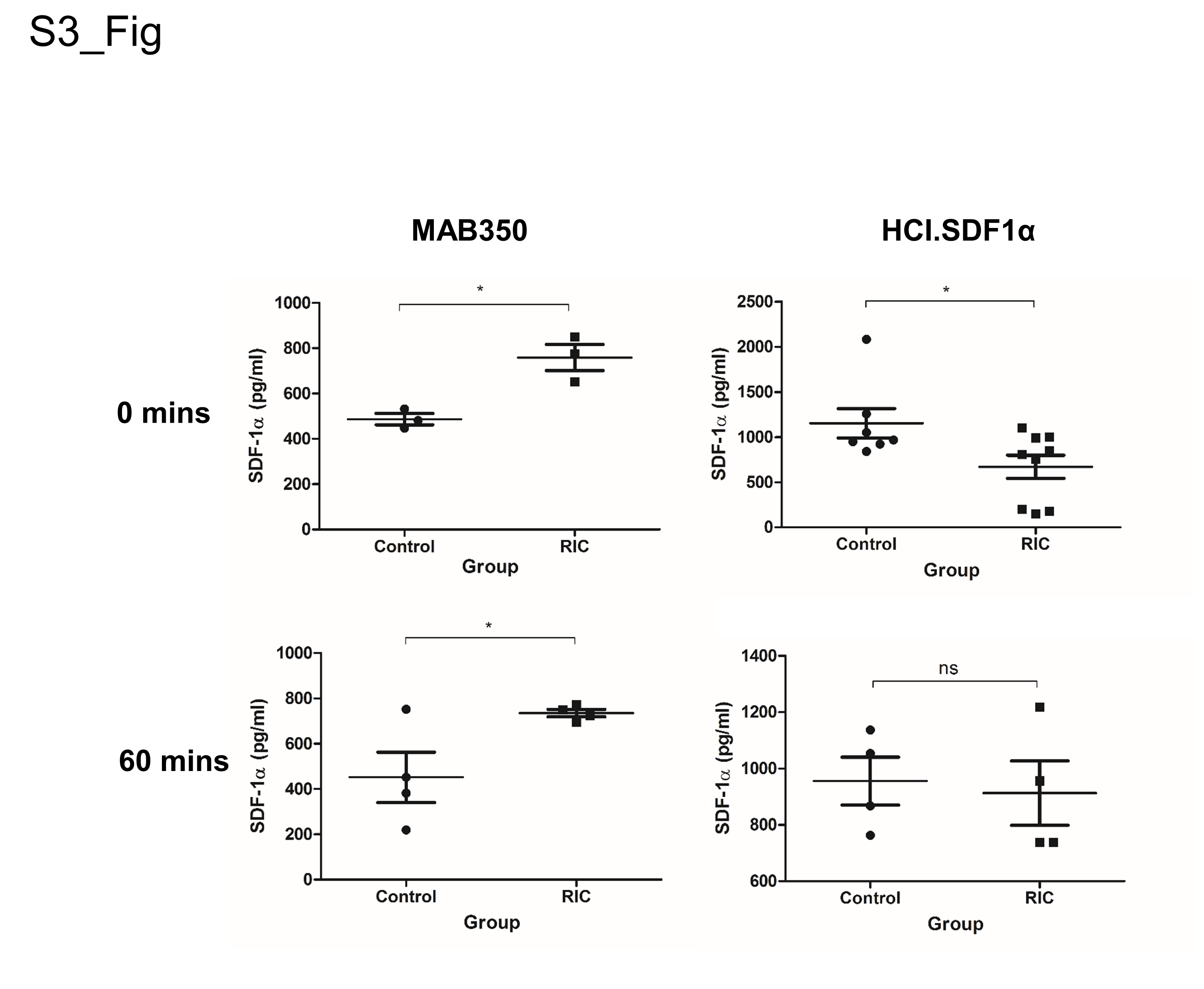

Supplement: S3 Fig — (TIF) [file pone.0174447.s003.tif]
